# Flow Cytometry for the Analysis of α-Dystroglycan Glycosylation in Fibroblasts from Patients with Dystroglycanopathies

**DOI:** 10.1371/journal.pone.0068958

**Published:** 2013-07-22

**Authors:** Elizabeth Stevens, Silvia Torelli, Lucy Feng, Rahul Phadke, Maggie C. Walter, Peter Schneiderat, Ayad Eddaoudi, Caroline A. Sewry, Francesco Muntoni

**Affiliations:** 1 Dubowitz Neuromuscular Centre, UCL Institute of Child Health/Great Ormond Street Hospital for Children, London, United Kingdom; 2 UCL Institute of Neurology, London, United Kingdom; 3 Friedrich-Baur-Institute, Department of Neurology, Ludwig-Maximilians-University Munich, Germany; 4 Flow Cytometry Core Facility, Camelia Botnar Laboratories, UCL Institute of Child Health/Great Ormond Street Hospital for Children, London, United Kingdom; University of Edinburgh, United Kingdom

## Abstract

α-dystroglycan (α-DG) is a peripheral membrane protein that is an integral component of the dystrophin-glycoprotein complex. In an inherited subset of muscular dystrophies known as dystroglycanopathies, α-DG has reduced glycosylation which results in lower affinity binding to several extracellular matrix proteins including laminins. The glycosylation status of α-DG is normally assessed by the binding of the α-DG antibody IIH6 to a specific glycan epitope on α-DG involved in laminin binding. Immunocytochemistry and immunoblotting are two of the most widely used methods to detect the amount of α-DG glycosylation in muscle. While the interpretation of the presence or absence of the epitope on muscle using these techniques is straightforward, the assessment of a mild defect can be challenging. In this study, flow cytometry was used to compare the amount of IIH6-reactive glycans in fibroblasts from dystroglycanopathy patients with defects in genes known to cause α-DG hypoglycosylation to the amount in fibroblasts from healthy and pathological control subjects. A total of twenty one dystroglycanopathy patient fibroblasts were assessed, as well as fibroblasts from three healthy controls and seven pathological controls. Control fibroblasts have clearly detectable amounts of IIH6-reactive glycans, and there is a significant difference in the amount of this glycosylation, as measured by the mean fluorescence intensity of an antibody recognising the epitope and the percentage of cells positive for the epitope, between these controls and dystroglycanopathy patient fibroblasts (p<0.0001 for both). Our results indicate that the amount of α-DG glycosylation in patient fibroblasts is comparable to that in patient skeletal muscle. This method could complement existing immunohistochemical assays in skeletal muscle as it is quantitative and simple to perform, and could be used when a muscle biopsy is not available. This test could also be used to assess the pathogenicity of variants of unknown significance in genes involved in dystroglycanopathies.

## Introduction

The congenital muscular dystrophies (CMDs) are a heterogeneous group of autosomal recessive disorders with varying degrees of clinical severity, broadly characterised by progressive muscle degeneration, weakness, and often central nervous system involvement. The dystroglycanopathies are a subgroup of the CMDs characterised by aberrant α-dystroglycan (α-DG) glycosylation. They are caused by mutations in several genes involved in the glycosylation of α-DG; Protein O-mannosyltransferase [1] (*POMT1;* MIM 607423), Protein O-mannosyltransferase 2 [2] (*POMT2;* MIM 607439), Protein O-mannose ß-1,2-N-acetylglucosaminyltransferase [3] (*POMGNT1;* MIM 606822), Fukutin [4] (*FKTN;* MIM 607440), Fukutin-related protein [5] (*FKRP;* MIM 606596), like-acetylglucosaminyltransferase [6] *(LARGE*; MIM 603590), Dolichyl-phosphate mannosyltransferase 2 [7] *(DPM2*: MIM **603564),** Dolichyl-phosphate mannosyltransferase 3 [8] *(DPM3*; MIM 605951), Dolichol Kinase [9] (*DOLK*; MIM 610746), Isoprenoid Synthase Domain Containing [10], [11], [12] (*ISPD*; MIM 614631), Glycosyltransferase-like domain containing 2 [13] (*GTDC2*; MIM 147730), β-1,3-N-acetylgalactosaminyltransferase 2 [14] (*B3GALNT2*; MIM 610194), Transmembrane protein 5 (*TMEM5*; MIM 605862) [15], β-1,3-N-acetylglucosaminyltransferase 1 (*B3GNT1*; MIM 605517) [16], GDP-mannose pyrophosphorylase B (*GMPPB*) [17], and protein kinase-like protein SgK196 (*SGK196*) [18].

Mutations in these genes account for the majority of dystroglycanopathy patients although not all carry mutations in any of these genes [10], [19], [20]. Primary dystroglycanopathy affecting the dystroglycan encoding gene (*DAG1*) itself is very rare and has only been described in one patient thus far [21]. The most severe forms of dystroglycanopathy are characterised by structural brain and ocular involvement, in additional to muscle degeneration and include Walker-Warburg syndrome (WWS, OMIM 236670), Muscle-Eye-Brain disease (MEB, OMIM 236670), and Fukuyama Congenital muscular dystrophy (FCMD, OMIM 253800). Milder forms include limb-girdle muscular dystrophies (LGMD) with no central nervous system involvement (e.g. LGMD2I, OMIM 607155) and intermediate forms include muscular dystrophies with or without intellectual disability such as congenital muscular dystrophy type 1C (MDC1C, OMIM 606612). This phenotypic classification system is described in more detail in Godfrey et al, 2011 [22].

In skeletal muscle, dystroglycan is encoded by a single gene and cleaved into α-DG and β-dystroglycan (β-DG) [23], [24], which bind non-covalently to one another and serve as a transmembrane linker within the dystrophin-glycoprotein complex (DGC), connecting the extracellular matrix (ECM) to dystrophin and the actin cytoskeleton. This connection is established by α-DG binding to C-terminal globular laminin-G (LG) domain containing proteins of the ECM (such as laminin-α2, agrin, perlecan, neurexin, pikachurin, and Slit [23], [25], [26], [27], [28], [29]) while β-DG binds to dystrophin and its homologue utrophin [30]. A specific *O*-mannose initiated glycan epitope within the mucin-like region of α-DG is thought to bind to these LG-domain containing ECM proteins. The composition of this glycan epitope has not yet been fully elucidated. The anti-α-DG IIH6 antibody has been found to recognise this epitope [24], [31] and blocks the interaction between α-DG and laminin-α2 [31]. The antibody has served as a useful diagnostic tool for the secondary dystroglycanopathies, allowing the extent of α-DG hypoglycosylation to be assessed by techniques such as immunohistochemistry and immunoblotting.

α-DG is highly glycosylated in a tissue specific and developmentally regulated way essential for its function [32], [33]. The core peptide is 74 kDa, but on SDS-PAGE gels it appears with an apparent molecular mass of 156 kDa in skeletal muscle and 120 kDa in brain and peripheral nerve due to extensive glycosylation in the mucin-like region of the protein [24]. Aberrant glycosylation of α-DG disrupts its connection with the ECM proteins described above, leading to weakened structural integrity of basement membranes in muscle, eye and brain, responsible for the characteristic pathology observed in these target tissues [34], although signalling defects could also play a role [34], [35].

Dystroglycan has been found to be expressed not only in muscle, eye and brain, the target organs in dystroglycanopathies, but also in a number of non-muscle tissues, including the kidney, liver and epithelia [32], [33], [36]. In skin, dystroglycan is present at the epidermal basement membrane zone [36]. In these tissues dystroglycan may serve as a link between epithelial cells and the basement membrane [36]. Dystroglycan in skin also binds to LG-domain containing ECM proteins, although its affinity to particular ligands is different to that of skeletal muscle dystroglycan, and its binding to perlecan is five times stronger than to the most active laminin fragment α-2LG1-3 [37], [38]. Additionally, laminin-α2 is reported to localise within the cytoplasm of basal keratinocytes in skin [39], rather than the basement membrane as is the case with muscle and peripheral nerve. The glycosylation of α-DG in dermal fibroblasts is also distinct from skeletal muscle and immunoblot analysis indicates that dermal α-DG appears as a broad smear with a molecular weight of approximately 120 kDa [36], [40].

In this study flow cytometry was used to assess the amount of IIH6-reactive glycans in dystroglycanopathy patient fibroblasts. We demonstrate that patients with a dystroglycanopathy consistently have lower levels of these glycans compared to healthy and pathological controls, and that the level in patient fibroblasts can be compared to that in skeletal muscle, despite tissue specific differences in glycosylation [36]. We suggest that this technique can be used as an experimental and diagnostic aid as it is quantitative and capable of detecting minor reductions in α-DG glycosylation.

## Materials and Methods

### Ethics Statement

All patient samples were obtained from the MRC CNMD Biobank London (REC reference number 06/Q0406/33). For all samples collected by the Biobank after 01/09/2006, written consent for research has been supplied by all patients or their parent/guardian if the participant was a child/minor. Skin biopsies were taken from patients and from adults with no known neuromuscular disease after obtaining written informed patient consent. All samples have been supplied to the ethically approved project on congenital muscular dystrophy [Ethics number 00/5802, reviewed by the Institutional Ethical Review Board of Great Ormond Street Hospital in the United Kingdom] anonymised. Copies of the consent forms are in the patient’s hospital notes.

### Subjects and Clinical Features

A total of twenty one dystroglycanopathy patient fibroblasts were assessed in this study; six with mutations in *FKRP*, three in *POMGNT1*, four in *POMT1*, two in *POMT2*, two in *ISPD*, two in *B3GALNT2,* and two in *GMPPB*. Fibroblasts from three healthy controls and seven pathological controls were also included in the study. The dystroglycanopathy patients included in this study included seven phenotypically LGMD patients, six MEB, two MDC1C, one with CMD (with no mental retardation), one with overlapping features of CMD and LGMD (with eye and brain involvement), and four with overlapping features of MEB and FCMD.

### Skin Biopsy and Fibroblast Culture

Fibroblasts were grown from skin biopsies and cultured in Dulbecco’s modified Eagles medium (Life Technologies, UK) supplemented with 20% fetal bovine serum (FBS, PAA, UK), 2% l-glutamine (Sigma, UK) and 1% penicillin, streptomycin and neomycin (Sigma, UK). Cells were cultured at 37°C in 5% CO_2_.

### Skeletal Muscle Histology and Immunohistochemistry

Immunohistochemichemical studies were performed as previously described [41]. Unfixed frozen serial sections (7 µm) were incubated with primary antibodies for 1 hour followed by three washes in phosphate buffered saline (PBS) pH 7.2, incubated with the appropriate biotinylated secondary antibodies for 30 minutes, and subsequently with streptavidin conjugated to Alexa 594 (Life technologies, UK) for 15 minutes. The primary antibody used to assess α-DG glycosylation was mouse monoclonal α-dystroglycan IIH6 (clone IIH6C4, from Kevin Campbell) [24], , α-dystroglycan VIA4-1 (Merck Millipore, UK). An antibody to β-dystroglycan (Leica, UK) was used as a control. Sections were evaluated using a Leica DMR microscope interfaced to MetaMorph (Molecular Devices, USA).

### Flow Cytometry Analysis of Patient Fibroblasts

The following method was modified from Rojek et al [42]. A few separate batches of the anti-α-DG antibody IIH6 (Merck Millipore, UK) was used to assess the amount of α-DG glycosylation in patient fibroblasts. Fibroblasts, below passage 10 whenever possible, were grown until approximately 90% confluent. Cells were detached using non-enzymatic cell dissociation solution in PBS (Sigma, UK), centrifuged for three minutes at 500 g, and counted and resuspended in PBS to a final density of 200,000 fibroblasts per millilitre. Cells were fixed with 2% paraformaldehyde for 10 minutes and washed with PBS supplemented with 0.1% FBS, centrifuged at 3,000 g for three minutes and incubated on ice with the following antibodies: anti-α-DG IIH6 or anti-α-DG VIA4-I for validation studies (Merck Millipore, UK) for thirty minutes, anti-mouse biotinylated IgM or IgG (Vector Labs, USA) for twenty minutes, and streptavidin-PE (BD Pharmingen, UK) for fifteen minutes**.** After washing, cells were re-suspended in 500 µl of PBS and transferred to FACS tubes (BD Biosciences, UK). Data was acquired using the Cyan ADP analyser (Beckman Coulter, USA) and analysed using the FlowJo software (Tree Star, USA). Two separate controls for each fibroblast population were used; one without any staining to remove background and another without the primary antibody to gate the IIH6 or VIA4-1 positive population. A total of 10,000 cells were analysed per experiment. Statistical analysis was performed using unpaired two-tailed *t*-tests where p<0.05 was considered significant. The amount of IIH6-reactive glycans was assessed by the number of cells producing the glycan epitope (the percentage of IIH6 positive cells) as well as by the level of IIH6 (the mean fluorescence intensity, or MFI). Additionally, the results were expressed using an integrated MFI value (iMFI) [43] [44], that combines the MFI of IIH6 and the percentage of IIH6 positive cells, and is defined by the following formula:
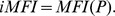
where *P* is the percentage of cells positive for the IIH6 epitope (table 1).

**Table 1:** Summary of α-DG glycosylation as assessed by flow cytometry in 21 patient fibroblasts, three healthy controls, and seven pathological controls.

ID	Gene	Mutation	Phenotype	MFI ± SEM	% IIH6 positive ± SEM	iMFI ± SEM	N	P (MFI)	P (% IIH6 positive)	P (iMFI)	Muscle α-DG IIH6 description
Control 1	n/a	Wild type	n/a	81.8±3.7	85.4±5.7	6437±435	12	N/A	N.A	N/A	Normal
Control 2	n/a	Wild type	n/a	77.8±5.4	83.7±6.2	7042±517	8	N/A	N/A	N/A	Normal
Control 3	n/a	Wild type	n/a	96.6±8.7	87.9±1.4	8110±752	8	N/A	N/A	N/A	Normal
Pathological control 1	*DYS*	Exon deletion: 52	DMD	75.6±2.3	70.0±2.3	5062±205	6	NS	0.006	0.013	Not done
Pathological control 2	*DYS*	Multiple exon deletion: 49–50	DMD	65.4±1.1	93.4±1.2	6113±102	6	0.01	NS	NS	Not done
Pathological control 3	*DYS*	Exon duplication: 3–4	DMD	63.3±4.3	95.1±0.7	6020±101	6	0.005	NS	NS	Not done
Pathological control 4	*DYS*	Exon 10 deletion (in frame)	BMD	94.4±3.2	84.7±2.4	8542±184	5	NS	NS	NS	Not done
Pathological control 5	*DYS*	Intron 2 splice site mutation	BMD	85.7±10.9	92.3±2.3	7812±796	5	NS	NS	NS	Not done
Pathological control 6	*RYR1*	homozygous p.S71Y	Myopathy	77.8±4.1	96.4±1.0	7466±379	6	NS	NS	NS	Normal
Pathological control 7	*GAA*	Genetic results unavailable; experiment result.	Glycogen storage disease II	77.1±2.6	72.3±0.50	5575±213	5	NS	0.01	NS	Not done
Patient 1	*FKRP*	heterozygous c.88C>T (p.Gln30X) and c.826C>A (p.Leu276Ile)	LGMD2I	53.3±3.5	44.6±4.7	2361±262	6	0.0001	<0.0001	<0.0001	Reduced
Patient 2	*FKRP*	homozygous c.1364C>A (p.Ala455Asp)	MEB	22.9±0.9	4.5±0.5	106±10	6	<0.0001	<0.0001	<0.0001	Absent
Patient 3	*FKRP*	homozygous 1023G>A (p.Trp341X)	MDC1C	39.9±3.3	26.3±1.5	1041±86	5	<0.0001	<0.0001	<0.0001	Not done
Patient 4	*FKRP*	heterozygous c.649C>A (p.Pro217Thr), c.1416G>T (p.Lys472Asn)	MDC1C	25.6±2.1	23.4±3.0	578±50	8	<0.0001	<0.0001	<0.0001	Markedly reduced
Patient 5	*FKRP*	homozygous c.826C>A (p.Leu276Ile)	LGMD2I	99.3±4.4	65.5±3.0	6380±256	5	NS	0.0001	NS	Reduced*
Patient 6	*FKRP*	homozygous c.826C>A (p.Leu276Ile)	LGMD2I	97.3±2.2	62.4±1.9	6150±240	5	NS	<0.0001	NS	Reduced*
Patient 7	*POMGNT1*	homozygous deletion c.33_34delGCinsA (p.Phe13fs)	MEB	20.5±1.6	8.1±0.8	190±34	6	<0.0001	<0.0001	<0.0001	Markedly reduced
Patient 8	*POMGNT1*	heterozygous c.1325G>A (p.Arg442His) and c.1582G>A (p.Val528Ile)	MEB	40.4±1.8	25.0±1.2	981±76	7	<0.0001	<0.0001	<0.0001	Reduced
Patient 9	*POMGNT1*	homozygous c.1342G>C (p.Gly448Arg)	MEB	22.9±1.9	20.2±6.8	474±84	6	<0.0001	<0.0001	<0.0001	Markedly reduced
Patient 10	*POMT1*	homozygous c.2179-2180delTC (p.Ser727fs)	MEB	29.0±3.5	15.1±1.3	430±47	6	<0.0001	<0.0001	<0.0001	Markedly reduced
Patient 11	*POMT1*	heterozygous c.598G>C (p.Ala200Pro) and c.2164G>A (p.Gly722Arg)	CMD (no MR)	51.1±3.3	41.6±3.9	2138±201	6	<0.0001	<0.0001	<0.0001	Reduced
Patient 12	*POMT1*	heterozygous c.598G>C (p.Ala200Pro) in exon 7 and c.427G>T (p.Glu143X)	LGMD2K	31.4±1.6	6.6±1.2	318±38	6	<0.0001	<0.0001	<0.0001	Markedly reduced
Patient 13	*POMT1*	heterozygous c.1958C>T (p.Pro653Leu) and c. 1241-2 A>G intron 12	LGMD2K	49.3±6.8	22.1±2.5	1060±155	6	0.0001	<0.0001	<0.0001	Normal
Patient 14	*POMT2*	heterozygous c.2047A>C (p.Thr683Pro) and c.1051delG (p.Ala351fs)	MEB-FCMD	36.5±2.0	32.2±4.4	1174±151	6	<0.0001	<0.0001	<0.0001	Markedly reduced
Patient 15	*POMT2*	homozygous c.661T>A (p.Phe211Ile)	MEB	49.4±2.4	59.2±3.2	2927±199	6	<0.0001	<0.0001	<0.0001	Not done
Patient 16	*ISPD*	heterozygous c.1183A>T (p.Arg395*) and c.1114-1116delGTT	LGMD (no MR)	38.2±3.0	33.6±7.0	1516±177	6	<0.0001	<0.0001	<0.0001	Markedly reduced
Patient 17	*ISPD*	homozygous duplication of ISPD exons 6,7, and 8	LGMD-CRB	48.9±2.5	7.9±0.4	386±29	6	<0.0001	<0.0001	<0.0001	Absent
Patient 18	*B3GALNT2*	heterozygous c.740G>A p.(Gly247Glu) and c.875G>C p.(Arg292Pro)	MEB/FCMD-like	33.5±3.4	14.1±1.8	765±112	6	<0.0001	<0.0001	<0.0001	Reduced
Patient 19	*B3GALNT2*	homozygous c.51_73dup p.(Ser25Cysfs*38)	MEB/FCMD-like	28.5±2.6	31.9±5.8	908±164	6	<0.0001	<0.0001	<0.0001	Reduced
Patient 20	*GMPPB*	heterozygous c.220C>T (p.Arg74X) and c.1000G>A (p.Asp334Asn)	MEB/FCMD-like	26.6±0.5	26.8±3.9	711±97	5	<0.0001	<0.0001	<0.0001	Reduced
Patient 21	*GMPPB*	heterozygous c.64C>T (p.Pro22Ser) and c.1000G>A (p.Asp334Asn)	LGMD (MR)	34.7±4.8	15.8±2.0	519±43	5	<0.0001	<0.0001	<0.0001	Reduced

The specific gene, mutation, phenotype, MFI of the IIH6 positive cells ± SEM, percentage of cells positive for the IIH6 epitope ± SEM, iMFI value ± SEM, N value, P value, and muscle α-DG IIH6 description is listed for each fibroblast cell line analysed. P values are the result of an unpaired t-test comparing the MFI, percentage of cells positive for the IIH6 epitope, or iMFI values of each fibroblast cell line to the respective values of the three healthy controls (C1, C2, C3). Muscle α-DG IIH6 description is the summary from the patient report of how the skeletal muscle α-DG glycosylation appeared by immunohistochemistry. iMFI is defined as iMFI = (MFI)(*P*), where P is the percentage of cells positive for the IIH6 epitope [43] [44]. Abbreviations are as follows: DMD, Duchenne muscular dystrophy; BMD, Becker muscular dystrophy; LGMD, Limb-girdle muscular dystrophy; MEB, Muscle-eye-brain disease; MDC1C, Muscular dystrophy type 1 C; CMD, Congenital muscular dystrophy; FCMD, Fukuyama congenital muscular dystrophy; LGMD-CRB, limb girdle muscular dystrophy with cerebellar involvement [11]; MR, mental retardation; NS, non-significant. * = Sections evaluated with the VIA4-1 antibody.

## Results

### Antibody Choice and Passage Number for Flow Cytometry Analysis of α-DG Glycosylation in Fibroblasts

The majority of previous studies on α-DG glycosylation have relied on using one of the antibodies which recognise the glycosylated epitopes of α-dystroglycan (namely the commercially available monoclonal antibodies IIH6 and VIA4-1) [45], [46], [47]. When assessing the extent of α-DG glycosylation using the commercially available antibodies, muscle immunohistochemistry is sensitive to batch-to-batch variation [41] yielding variable results. This batch variation also results in reduced performance for some antibodies/batches over others using standard diagnostic techniques.

For flow cytometry, however, different batches of the commercially available antibody anti-α-DG IIH6 (Merck Millipore, UK) as well as anti-α-DG VIA4-I (Merck Millipore, UK) yielded consistent results with non-significant differences in MFI values or the percentage of cells positive for the IIH6 epitope for all four fibroblast cell lines tested with multiple antibodies/batches. Control 1 (C1) was tested with two batches of IIH6 and one batch of VIA4-1 and the MFI values as well as the percentage of cells positive showed no significant change regardless of batch or antibody choice (data not shown). C2, pathological control 1 (PC1), patient 18 (P18), and P19 were also tested with two or more batches of IIH6 and VIA4-1 and also showed no significant variation in either of the values when using different primary anti-α-DG antibodies/batches (data not shown). This may be partially due to the fact that with this method the cells are detached prior to analysis, so cell-cell and cell-matrix interactions do not impair antibody binding to the IIH6 and VIA4-1 glycan epitopes.

In order to assess the effect that different passage number had on the outcome of the results, we performed a series of relevant experiments. These showed that when the fibroblasts were tested at later passages (>passage 10) a reduction in the MFI of IIH6-reactive glycans was observed in control and patient fibroblast cell lines. Specifically, the MFI value of C1 at passage 5 and C2 at passage 6 was significantly (p<0.0001) higher than C1 at passage 10 and C2 at passage 11. An additional DMD pathological control was also tested at a later passage (14) and had a MFI of 31.3 (n = 3), a significant reduction compared to the DMD cases PC1, PC2 and PC3 as well as C1, C2, and C3 (when below passage 10) (p<0.0001 in all cases)(data not shown). It should therefore be noted that passage number should be strictly standardised for reproducible and valid results using this method. Our results indicate that a passage number of <10 should be used.

### Assessment of α-DG Glycosylation in Healthy and Pathological Control Fibroblasts

To ensure the validity of this technique, fibroblast cell lines from three healthy controls and seven pathological controls were first tested (table 1). The pathological controls included 5 fibroblast cell lines with mutations in the dystrophin gene (*DYS*, MIM 300377; PC 1, 2, 3, 4, 5), one fibroblast cell line with a ryanodine receptor mutation (*RYR1*, MIM 180901; PC6), and one fibroblast cell line with a mutation in the α-glucosidase gene (*GAA,* MIM 232300; PC7). They were individually screened for the amount of IIH6-reactive glycans by flow cytometry.

All healthy control fibroblast cell lines and pathological control cell lines were between 70 and 96.4% positive for IIH6-reactive glycans (table 1). The healthy control fibroblasts (C1, C2, C3) had an average MFI value of 85.4±5.7 and the pathological control fibroblasts had an average MFI value of 77.0±4.1 (not significantly reduced). Individually, all of the pathological controls had a non-significant difference in MFI compared to the healthy controls, with the exception of the Duchenne muscular dystrophy (DMD) fibroblasts PC2 and PC3. PC2 and PC3 showed a significant reduction in their level of IIH6-reactive glycans compared to control fibroblasts with a MFI of 65.4 (p = 0.01) and 63.3 (p = 0.005), however the percentage of cells positive for the IIH6 epitope for both patients were not significantly reduced compared to healthy controls. Additionally, while PC1 had a non-significant reduction in MFI compared to controls, the cells had a significantly reduced percentage of cells positive for IIH6 (p = 0.006). The significant reduction in MFI for PC2 and PC3 as well as the percentage of IIH6 positive cells for PC1 may be due to the fact that dystrophin is expressed at low levels in fibroblast cultures [48], [49], possibly leading to a perturbation in the DGC. The two Becker muscular dystrophy (BMD) patients tested (PC4, PC5) had no reduction in MFI or the percentage of cells positive for the IIH6 epitope, however. DMD patients are clinically and pathologically distinct from suspected dystroglycanopathy patients, so this should not limit the diagnostic efficacy of this technique.

### The Amount of Glycosylated α-DG in Dystroglycanopathy Patient Fibroblasts is Consistently Reduced Compared to Healthy Control Fibroblasts

Fibroblasts from 21 dystroglycanopathy patients were tested for α-DG glycosylation by flow cytometry (table 1). By skeletal muscle immunohistochemistry, β-dystroglycan and core α-DG was normal for all patients and controls studied (data not shown). All patient fibroblasts tested had a significantly reduced percentage of cells positive for the epitope compared to healthy controls as well as a significantly reduced MFI, with the exception of the two LGMD2I patient fibroblasts (P5 and P6, table 1) homozygous for c.826C>A (p.Leu276Ile) in *FKRP*.

**Figure 1:**
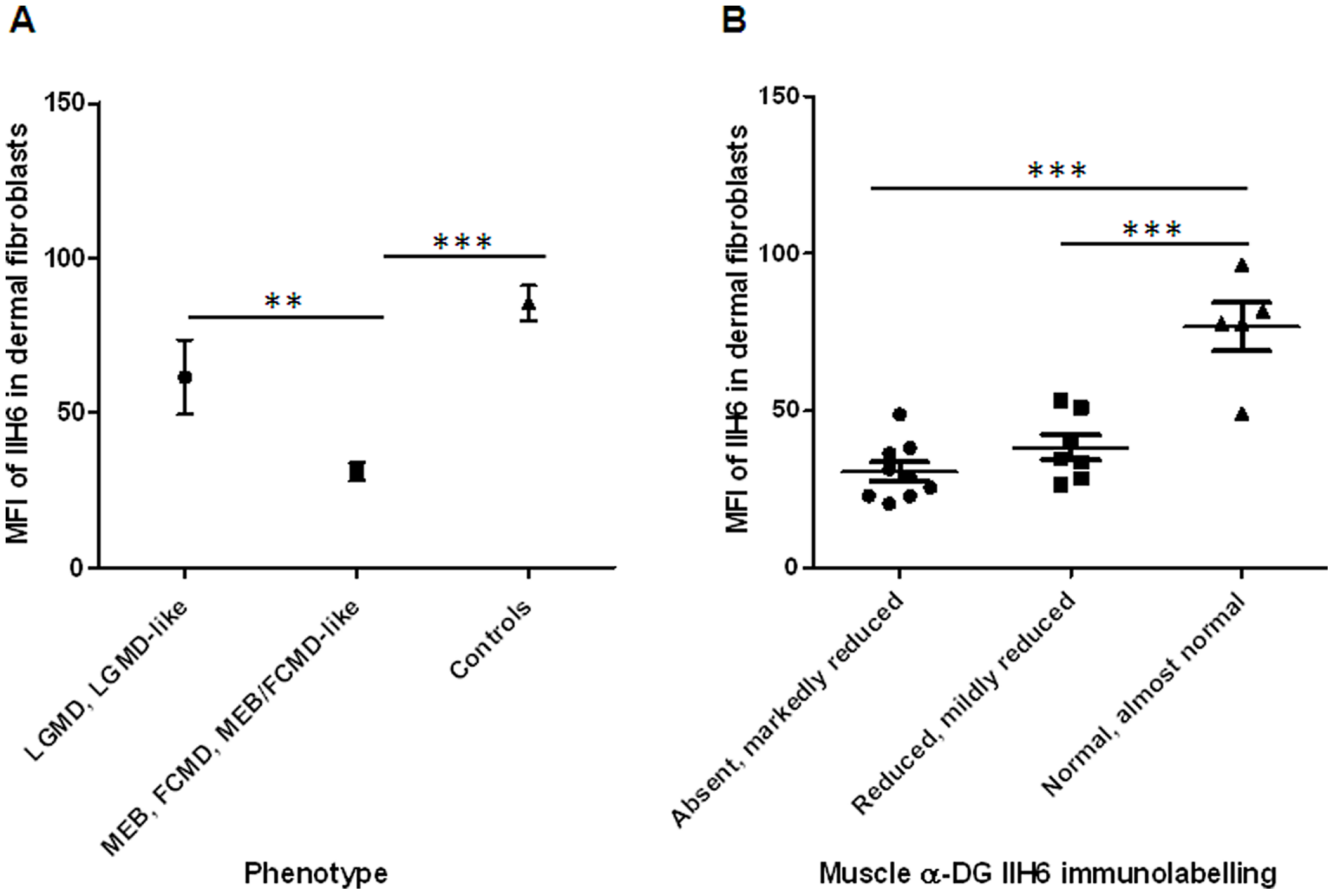
Comparison of the mean fluorescence intensity (MFI) of α-DG glycosylation in patient fibroblasts to respective patient phenotypic severity and skeletal muscle α-DG IIH6 immunolabelling. (A) Fibroblasts from patients with relatively mild phenotypes (LGMD, LGMD-like without brain involvement, includes P1, P5, P6, P12, P13, and P16 from table 1) have a significantly (p = 0.008) higher average MFI value (61.5±12.1) than fibroblasts from patients with more severe dystroglycanopathy phenotypes (MEB, FCMD, MEB/FCMD-like, includes P2, P7, P8, P9, P10, P14, P15, P18, P19, P20 with an MFI average of 31.0±2.9). (B) In patient fibroblasts, a decrease in the MFI of IIH6 obtained by flow cytometry concomitant with a decrease in the intensity of immunolabelling in skeletal muscle sections was observed. Skeletal muscle sections which were described as ‘absent’ or ‘markedly reduced’ (table 1) for IIH6 yielded an average MFI from the respective fibroblasts of 30.7±3.1 (n = 9). Sections described as ‘reduced’ for IIH6 yielded an average MFI value of 38.3±4.0 (n = 7) from the respective fibroblasts. Finally, sections which were described as ‘normal’ (or almost normal) yielded MFI values of 76.7±7.7 (n = 5) from the respective fibroblasts. There is a significant difference in the MFI between both groups reduced for skeletal muscle α-DG IIH6 immunolabelling and the group described as normal or almost normal (p<0.0001 for absent/markedly reduced, p = 0.0007 for reduced). *** = p<0.001, ** = p<0.01 (unpaired t-test). In all cases values are described as mean fluorescence intensity ± standard error of the mean (SEM).

Flow cytometry of dystroglycanopathy patient fibroblasts revealed that even severely affected patient fibroblasts (e.g. MEB/FCMD) have detectable, albeit significantly reduced levels of α-DG glycosylation. Fibroblasts from patients with MEB, FCMD, MEB/FCMD-like phenotypes (e.g. P2, P7, P8, P9, P10, P14, P15, P18, P19, P20) had an average MFI of 31.0±2.9 (figure 1A), statistically reduced compared to the MFI values of the healthy controls (C1, C2, C3, 85.4±5.7, P<0.0001). P7 has a MEB phenotype (mutations in *POMGNT1*) and a 76% reduction in MFI compared to the average MFI value of healthy controls (an MFI value of 20.5 compared to an average MFI value of 85.4). Additionally, P7 has a 90.5% reduction in the percentage of IIH6 positive cells compared to healthy controls (8.1% positive compared to an average of 85.7% positive). She was hypotonic and weak from birth, and at the age of 16 months had abnormal eye movements and no head control. Less severely affected patient fibroblasts (e.g. LGMD, LGMD-like) generally have a less but nonetheless significant reduction in MFI. Patients with LGMD or LGMD-like phenotypes with no brain involvement (e.g. P1, P5, P6, P12, P13, and P16) had an average MFI of 61.5±12.1 which is significantly higher than that of MEB/FCMD patients (p = 0.008, figure 1A). There was a non-significant difference in the average percentage of IIH6 positive cells in LGMD and LGMD-like patient fibroblasts compared to MEB/FCMD patient fibroblasts (39.1±9.4 compared to 23.7±4.9), suggesting that MFI may be a better outcome measure. P16 has a LGMD phenotype (mutations in *ISPD*) and a 55% decrease in MFI and a 60.8% decrease in IIH6 positive cells compared to healthy controls (an MFI of 38.2 and 33.6% cells positive for IIH6, table 1). P16 is currently 12 years of age and has followed a severe LGMD-like disease course; she is currently non-ambulant [11]. Previous studies on skeletal muscle [19] have found that patients with severe dystroglycanopathy phenotypes do not always have less α-DG glycosylation than milder phenotypes when assessed using immunohistochemical studies on skeletal muscle biopsies, especially for *FKRP* and *FKTN*, although there is a broad correlation for *POMT1*, *POMT2*, and *POMGNT1.*

### Flow Cytometry as a Complementary Diagnostic Method to Muscle Immunohistochemistry

**Figure 2:**
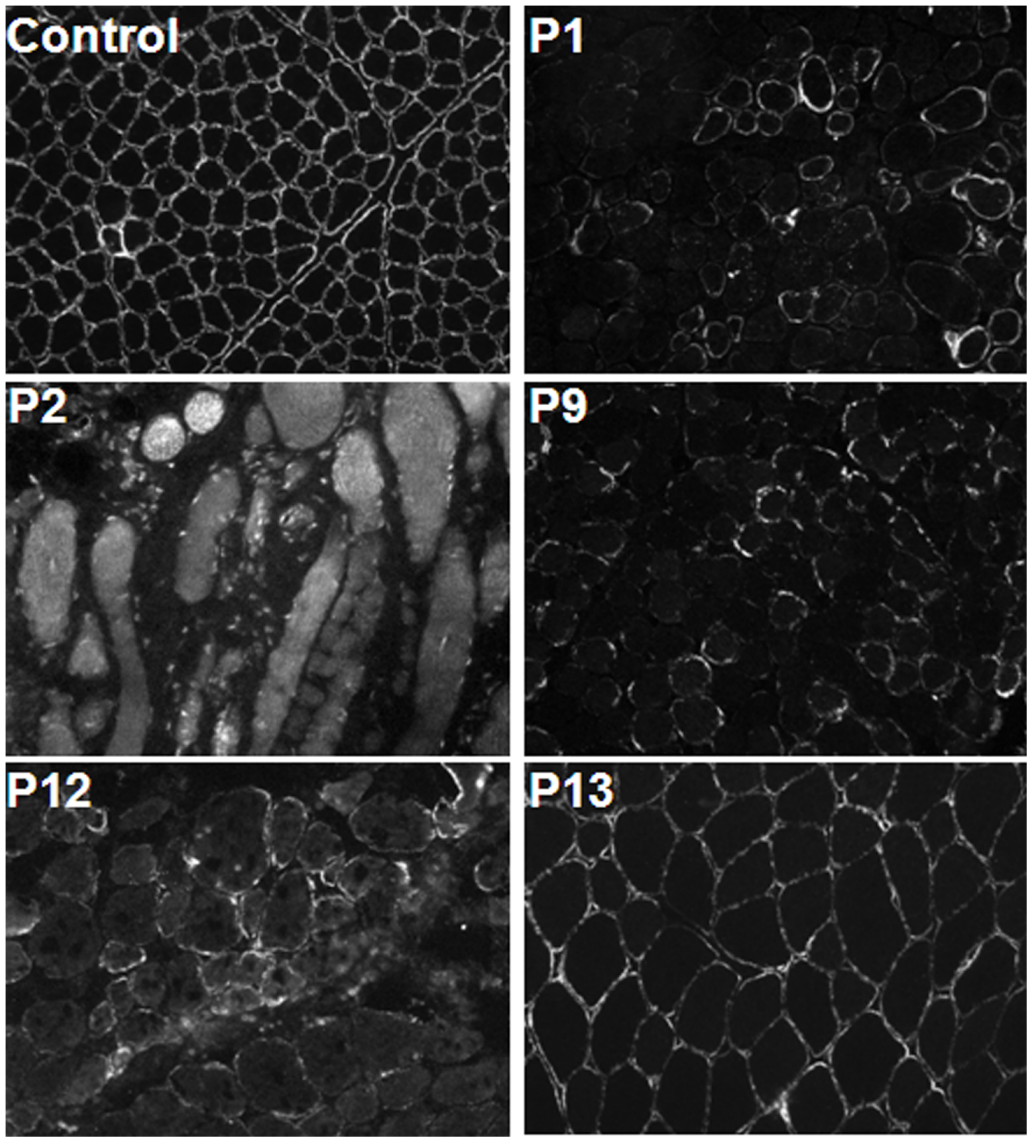
Skeletal muscle immunohistochemistry from patients with dystroglycanopathy mutations. Skeletal muscle cryo-sections from control and dystroglycanopathy patients P1, P2, P9, P12, and P13 immunolabelled with the anti-α-DG IIH6 antibody. P1 and P2 have mutations in *FKRP*, P9 has a mutation in *POMGNT1*, P12 and P13 have mutations in *POMT1*. β-dystroglycan and core α-DG was similar to controls for all patients (data not shown) but the amount of IIH6-reactive glycans varied markedly. In P1 the level of IIH6 reactive glycans was reduced, in P2 absent, in P9 and P12 markedly reduced, and in P13 normal (or near normal) compared to the healthy control.

The IIH6 staining in skeletal muscle of the dystroglycanopathy patients included in this study either showed normal (or almost normal), markedly reduced, reduced, or absent α-DG glycosylation (table 1). In figure 2 the patients have been divided into four categories based on skeletal muscle immunohistochemistry, as assessed by the diagnostic team’s routine service. Figure 2 illustrates some of the difficulties and subjectivity in interpreting the extent of α-DG glycosylation given the variable results of muscle immunohistochemistry with the anti-α-DG IIH6 antibody.

In fibroblasts there was a decrease in the MFI of IIH6 concomitant with a decrease in the intensity of immunolabelling in the respective patient skeletal muscle sections (figure 1B), despite the use of different antibodies for the detection of glycosylated α-DG and tissue specific differences in glycosylation between skeletal muscle and fibroblasts [36]. Skeletal muscle sections which were described as ‘absent’ or ‘markedly reduced’ (table 1) for IIH6 labelling yielded an average MFI value of 30.7±3.1 (n = 9) and an average percentage positive for IIH6 of 16.9±3.7. Sections described as ‘reduced’ for IIH6 yielded an average MFI value of 38.3±4.0 (n = 7) and an average percentage positive for IIH6 of 27.9±3.9. Finally, sections which were described as ‘normal’ (or almost normal) yielded an average MFI value of 76.7±7.0 (n = 5) and an average percentage positive for IIH6 of 75.1±13.4.

Similar to the results of previous studies on skeletal muscle [19], [47] there was no direct correlation between primary gene defect and the extent of α-DG hypo-glycosylation. P10–P13 all carry mutations in *POMT1*, and the extent of the reduction of glycosylated α-DG on skeletal muscle sections ranged from ‘markedly reduced’ to ‘almost normal’ and the MFI of IIH6 in patient fibroblasts were between 29.0 and 51.1 (normal control MFI values were between 77.8 and 96.6). These results highlight the fact that it is the severity of the individual gene defect that determines severity of phenotype, not necessarily the gene primarily involved.

## Discussion

The dystroglycanopathies encompass a wide range of clinical severities and variable levels of α-DG glycosylation defects. An ideal technique to assess patients (or animal models) affected by these conditions should ideally allow the detection of even mild reductions in α-DG glycosylation. This can be difficult to establish with the currently available methods. In this study we show that flow cytometry is suited for this task, as it allows us to quantitatively determine the extent of α-DG glycosylation in normal and dystroglycanopathy patient fibroblasts. Recent studies have also used flow cytometry as a complementary diagnostic technique for other proteins in which a deficiency causes muscular dystrophy. In the study by Kim et al [50], flow cytometry was used to quantitatively determine the amount of collagen VI expression in fibroblasts from patients with collagen VI myopathies.

The sensitivity of flow cytometry allows the detection of slight reductions in the level of IIH6 reactive glycans, which may be equivocal by immunohistochemistry. The skeletal muscle biopsy of P13 (figure 2) showed normal or almost normal levels of IIH6 reactive glycans as compared to control skeletal muscle. It was therefore difficult to determine whether this was a dystroglycanopathy case or not based on the results of the muscle pathology assessment alone. Flow cytometry of P13’s fibroblasts gave an MFI value of 49.3 with 22.1% positive for IIH6 reactive glycans, both significantly reduced compared to healthy controls (p = 0.0001 and p<0.0001, respectively). Sanger sequencing eventually revealed that this patient has mutations in *POMT1*. If flow cytometry had been used alongside immunohistochemistry, it would have immediately revealed that P13 was likely a dystroglycanopathy case.

The integrated MFI or iMFI was included in table 1 to represent the total amount of IIH6 detected in fibroblasts by flow cytometry by incorporating both the percentage of IIH6 positive cells and the MFI of those positive cells into a single, more sensitive outcome measure [43] [44]. While integrating both values into a single iMFI value does not give the same information as having both values individually, it provides a useful estimation of the total amount of IIH6. P17’s skeletal muscle was found to be completely absent for the IIH6 epitope by immunohistochemistry. The MFI of this patient, however, was only approximately 42% reduced compared to healthy controls (an MFI of 48.9 and 85.4, respectively) while the iMFI was almost 97% reduced (an iMFI of 385.6 and 7196, respectively). The iMFI therefore better corresponds to the marked reduction observed in P17’s skeletal muscle immunohistochemistry. This was similarly the case for P2, P12, P14, and P16. Additionally, in some cases iMFI better distinguishes dystroglycanopathy patients who are slightly reduced for IIH6 from pathological controls (table 1). There is a 29.2% reduction in MFI between PC1 and P1 (75.6 and 53.1, respectively) but a 53.4% reduction in iMFI between the two (5062 and 2361, respectively). Similar results were also observed for P8, P11, P13, P15, and P17. Based on these data, iMFI is better able to separate patients with mild reductions in IIH6 from pathological controls than MFI alone, and better correlates with immunohistochemical data.

This flow cytometry method can also be used as a tool to determine whether a patient has a dystroglycanopathy phenotype when a confirmed genetic diagnosis is not yet available. For example, when P18 and P19 with mutations in *B3GALNT2* and P20 and P21 with mutations in *GMPPB* were first analysed by flow cytometry, variants in these genes had not yet been identified as the cause of a dystroglycanopathy. In all four patient’s fibroblasts, flow cytometry revealed a reduction in the MFI of IIH6 and the percentage of cells positive for the epitope and this was comparable to the amount detected by skeletal muscle immunohistochemistry [14], [17]. Our group subsequently confirmed that variants in both *B3GALNT2* and *GMPPB* can cause dystroglycanopathy.

LGMD2I patients homozygous for the c.826C>A (p.Leu276Ile) variant in *FKRP* often have mild skeletal muscle pathology, making diagnosis based on muscle biopsy alone challenging [51]. Therefore, we hoped that flow cytometry would be sensitive enough to detect a reduction in the amount of IIH6-reactive glycans in fibroblasts from these patients. The fibroblasts were found to not be significantly reduced in the MFI of their IIH6 reactive glycans compared to healthy controls but were significantly reduced in the percentage of cells positive for IIH6 (p = 0.0001 for P5 and <0.0001 for P6, table 1). Both patients were reduced for VIA4-1 reactivity by skeletal muscle immunohistochemistry. More LGMD2I patient fibroblasts with the homozygous mutation c.826C>A (p.Leu276Ile) would need to be studied to see if this reduction in the percentage of fibroblasts positive for IIH6 compared to healthy control fibroblasts is a consistent finding which could be used to aid diagnosis.

It should be reinforced that α-DG glycosylation is tissue specific [32], [33], [36], which may modulate its laminin-binding specificity [52]. Consequently, the results of this study do not allow us to infer the level of α-DG glycosylation in muscle directly. Additionally, there are limitations in interpreting the amount of α-DG glycosylation based solely on the IIH6 epitope, as other unknown glycan epitopes on α-DG may also have significance for the function of this receptor in muscle. Nonetheless, we were able to demonstrate that patients with reduced IIH6 immunolabelling on skeletal muscle sections also had similarly reduced MFI values for IIH6 in fibroblasts as assessed by flow cytometry. Larger studies will be required in order to confirm the precise correlation between α-DG glycosylation on fibroblasts with skeletal muscle immunohistochemistry and clinical features. This study nevertheless clearly indicates that analysis of dystroglycan glycosylation in fibroblasts is a useful diagnostic aid for the dystroglycanopathies, as flow cytometry allows us to detect changes in α-DG glycosylation. It can also be used to identify possible dystroglycanopathy cases where the muscle immunohistochemistry result is equivocal or the genetic etiology is not known.
